# Chemical genomic analysis reveals the interplay between iron chelation, zinc homeostasis, and retromer function in the bioactivity of an ethanol adduct of the feijoa fruit–derived ellagitannin vescalagin

**DOI:** 10.1093/g3journal/jkae098

**Published:** 2024-05-28

**Authors:** Mona Mokhtari, Pegah Amiri, Darach Miller, David Gresham, Stephen J Bloor, Andrew B Munkacsi

**Affiliations:** School of Biological Sciences, Victoria University of Wellington, Wellington 6012, New Zealand; School of Biological Sciences, Victoria University of Wellington, Wellington 6012, New Zealand; Department of Genetics, Stanford University Medical School, Stanford, CA 94305, USA; Department of Biology, Center for Genomics and Systems Biology, New York University, New York, NY 10003, USA; Department of Biology, Center for Genomics and Systems Biology, New York University, New York, NY 10003, USA; Callaghan Innovation, Lower Hutt 5040, New Zealand; School of Biological Sciences, Victoria University of Wellington, Wellington 6012, New Zealand; Centre for Biodiscovery, Victoria University of Wellington, Wellington 6012, New Zealand

**Keywords:** chemical genetics, iron, zinc, retromer, yeast, ellagitannin, bioactivity

## Abstract

Nature has been a rich source of pharmaceutical compounds, producing 80% of our currently prescribed drugs. The feijoa plant, *Acca sellowiana*, is classified in the family Myrtaceae, native to South America, and currently grown worldwide to produce feijoa fruit. Feijoa is a rich source of bioactive compounds with anticancer, anti-inflammatory, antibacterial, and antifungal activities; however, the mechanism of action of these compounds is largely not known. Here, we used chemical genetic analyses in the model organism *Saccharomyces cerevisiae* to investigate the mechanism of action of a feijoa-derived ethanol adduct of vescalagin (EtOH-vescalagin). Genome-wide barcode sequencing analysis revealed yeast strains lacking genes in iron metabolism, zinc metabolism, retromer function, or mitochondrial function were hypersensitive to 0.3 µM EtOH-vescalagin. This treatment increased expression of iron uptake proteins at the plasma membrane, which was a compensatory response to reduced intracellular iron. Likewise, EtOH-vescalagin increased expression of the Cot1 protein in the vacuolar membrane that transports zinc into the vacuole to prevent cytoplasmic accumulation of zinc. Each individual subunit in the retromer complex was required for the iron homeostatic mechanism of EtOH-vescalagin, while only the cargo recognition component in the retromer complex was required for the zinc homeostatic mechanism. Overexpression of either retromer subunits or high-affinity iron transporters suppressed EtOH-vescalagin bioactivity in a zinc-replete condition, while overexpression of only retromer subunits increased EtOH-vescalagin bioactivity in a zinc-deficient condition. Together, these results indicate that EtOH-vescalagin bioactivity begins with extracellular iron chelation and proceeds with intracellular transport of zinc via the retromer complex. More broadly, this is the first report of a bioactive compound to further characterize the poorly understood interaction between zinc metabolism and retromer function.

## Introduction

**Fig. 1.**
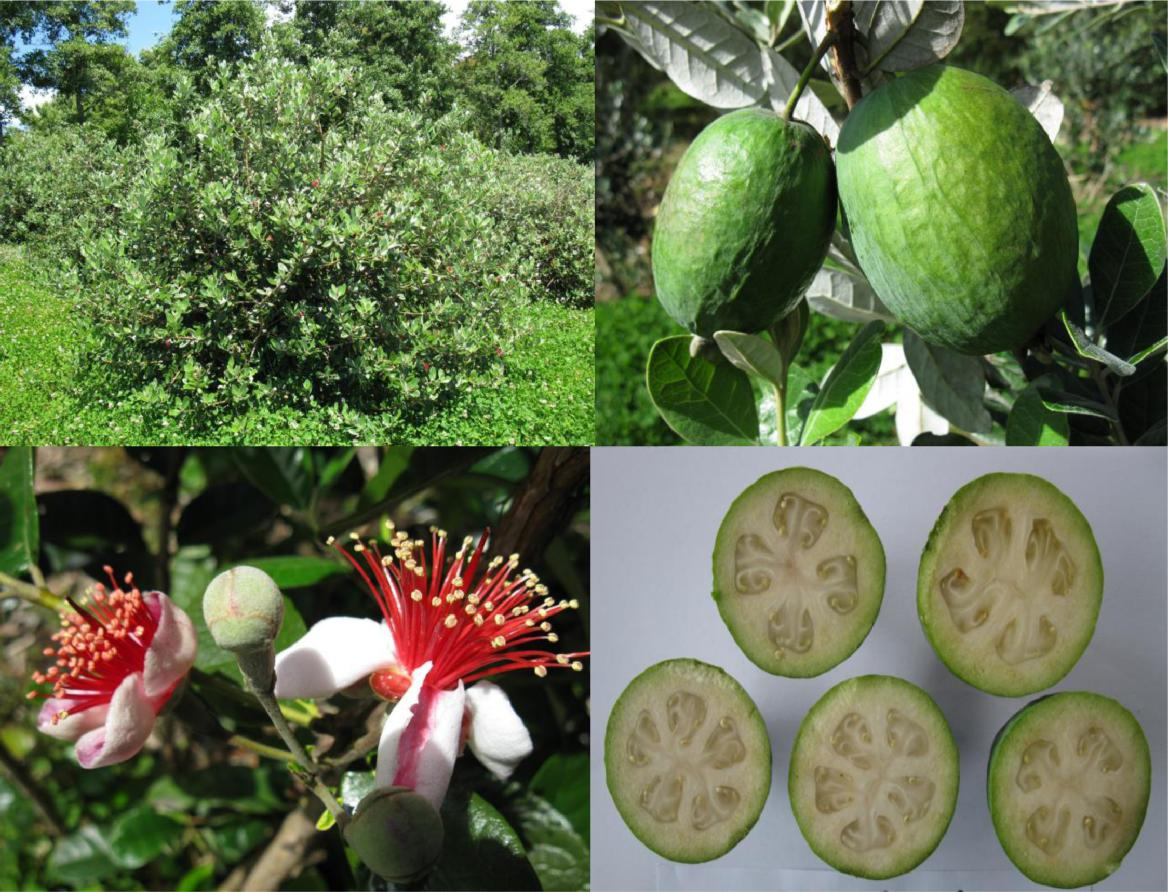
Representative images of feijoa including the whole plant, flowers, fruit, and cross-section of fruit. Reprinted with permission from Mokhtari *et al.* 2018; © 2018 American Chemical Society.

Feijoa is native to southern Brazil, northern Argentina, and western Paraguay where it is known as pineapple guava. It is actively cultivated as an agricultural crop in temperate and subtropical climates in the United States of America, South America, Europe, Australia, and New Zealand. The pulp of the fruit (Fig. 1) is consumed entirely or as a juice, as well as in food products such as ice cream, chutney, chocolate, and wine. The fruit pulp has a gritty texture with a distinctive highly perfumed odor and a flavor that is sweet to mid-sour. Bioactive compounds with anticancer, antioxidant, antiviral, anti-inflammatory, antibacterial, and antifungal activities have been isolated from the leaves, pulp, and peels of commercial cultivars of the thick-skinned feijoa fruit (*Acca sellowiana*, Myrtacaeae) (Weston 2010; Mokhtari *et al*. 2018; Zhu 2018; Phan *et al*. 2019; de Oliveira Schmidt *et al*. 2020; Montoro *et al*. 2020; Santos *et al*. 2021).

Tannins are a class of bioactive, polyphenolic compounds that exist in most plants with critical roles in plant growth and defense against microbial pathogens. The major classes of tannins are hydrolyzable tannins, condensed tannins, and phlorotannins. Hydrolyzable tannins are primarily either gallotannins with repeated units of gallic acid or ellagitannins with repeated units of ellagic acid. Both gallotannin and ellagitannin compounds have been reported with anticancer, antifungal, antibacterial, antiviral, antioxidant, and anti-inflammatory activities (Lipińska *et al*. 2014; He 2022). Recently, our institution developed an extraction method that yielded an extract of feijoa fruit enriched in water-soluble tannin polyphenols including castalagin, pedunculagin, pentagalloyl glucose, casuarinin, and an ethanol adduct of vescalagin (Foo and Watson 2018). It is now critical to elucidate the mechanism of action of these tannins in feijoa fruit to inform bioactivity of these compounds as pharmaceutical and/or nutraceutical agents, thus raising the potential for classifying feijoa fruit as a functional food.

In the event that the bioactivity and/or mechanism of action of an extract or compound is not known, chemical genetics is a reliable approach for uncovering the genes that mediate the bioactivity of a compound (Parsons *et al*. 2006). Molecular mechanisms of bioactive compounds have been determined using the genetic model Baker's yeast (*Saccharomyces cerevisiae*) by elucidating the relationship between genotype and phenotype via measurements of growth of gene deletion strains in genome-wide analyses, which are readily completed by measuring colony size on agar or quantifying abundance of 20-bp barcodes upstream (UPTAG) and downstream (DNTAG) of gene deletions. The genes buffering the mechanism of action of thousands of compounds have been determined by barcode sequencing (Bar-seq) analysis of gene deletion libraries (Hillenmeyer *et al*. 2008; Lee *et al*. 2014).

Here, we build off previous success of using chemical genetic analysis in yeast to identify mechanism of action of an antifungal compound in feijoa peels (Mokhtari *et al*. 2018). Using our established Bar-seq methodology (Robinson *et al*. 2014; Sun *et al*. 2020), we investigated bioactivity of polyphenol compounds in feijoa fruit, identified EtOH-vescalagin as the most bioactive, and defined its mechanism of action including iron depletion, zinc toxicity, and disrupted retromer transport.

## Materials and methods

### Strains and media

All strains of *S. cerevisiae* were obtained from Open Biosystems (Huntsville, AL, USA) in the haploid BY4741 background and diploid BY4743 background. Pathogenic *Candida* strains were obtained from American Type Culture Collection (Manassas, VA, USA) and included *Candida albicans* (ATCC 10231), *Candida glabrata* (ATCC 90030), *Candida parapsilosis* (ATCC 90018), and *Candida tropicalis* (ATCC 13803). Strains were grown in SC media buffered with 25 mM HEPES (hereafter referred to as SC) with dropout of specific amino acids as required for selection.

### Liquid-based assay of bioactivity

Fungal growth was measured in a liquid assay as previously described (Molimau-Samasoni *et al*. 2021). Fungal cultures at 5 × 10^5^ cells/mL were grown in SC at 30°C and quantified via absorbance at 600 nm using a PerkinElmer EnVision Plate Reader (PerkinElmer, Waltham, MA, USA) at a time point when the control was mid-log (OD_600_ = 0.4). Growth inhibition was calculated using the following formula:


$$\mathrm{Growth}\;\mathrm{inhibition}\;(\text{\%})=100\text{–}[(OD_{600}\;\mathrm{of}\;\mathrm{treatment}/OD_{600}\;\mathrm{of}\;\mathrm{control})\times 100].$$

### Genome-wide Bar-seq analysis

Sensitivity of the homozygous diploid deletion mutant library was evaluated using Bar-seq as previously described (Robinson *et al*. 2014). First, the diploid homozygous deletion library (Open Biosystems) was pooled to a density of 1 × 10^9^ cells/mL. A single aliquot (1 mL) of the same frozen stock of the diploid homozygous deletion library was then inoculated in 10 mL SC and incubated at 30°C for 18 h, and this culture was used to inoculate 100 mL SC at 5 × 10^6^ cells/mL that was further incubated at 30°C for 5 h. These cells in log phase were used to inoculate fresh 2 mL SC in the presence and absence of EtOH-vescalagin to a final concentration of 5 × 10^6^ cells/mL (i.e. ∼1,000 representations of each strain) and incubated at 30°C for 15 h (the first 10 generations), which was measured at OD_600_ to confirm that cells were at log phase (OD_600_ = 0.4–0.6) and to confirm EtOH-vescalagin reduced growth by 20% compared with untreated cells. Cells from the first 10 generations were then used to inoculate fresh 2 mL SC in the presence and absence of 305 nM EtOH-vescalagin to a final concentration of 5 × 10^6^ cells/mL and incubated at 30°C for 15 h (the second 10 generations). Then, genomic DNA (gDNA) was extracted from 5 × 10^7^ cells using the YeaStar Genomic DNA Kit (Zymo Research) according to protocol I in the manufacturer's instructions to yield 10–20 μg purified gDNA. gDNA was used as template in a PCR using universal primers to amplify the unique 20-bp UPTAG and 20-bp DNTAG barcodes representative of every gene deletion. Then, 60 ng of each UPTAG and DNTAG amplicon library pool separately underwent PCR amplification for the incorporation of the Illumina P5 adapter sequence, and finally, the UPTAG and DNTAG libraries were combined at a 1:1 ratio, and 50-bp single-end reads were sequenced on an Illumina flow cell using an Illumina HiSeq 2000 platform. Read matching and bioinformatic analyses were conducted as previously described to generate logFC (change in mutant barcode sequence abundance in treatment compared with control), false discovery rate, and logCPM (quality of Bar-seq results) for every gene deletion in the library. As we ran a triplicate treatment assay, for the final analysis on Bar-seq residual growth values, we ran growth reduction calculation for each deletion strain replicate, and we calculated residual growth for each treated vs untreated counterparts (treatment growth/untreated), so we had 3 values, and then, 2 out of 3 values < 0.5 were picked as top hits. Validation of top hit-sensitive strains in the competitive pool format was evaluated via growth analyses of specific strains (in standard isolated format) using the liquid growth assay.

### Gene annotation

Functional annotations and human orthologues of yeast genes were derived from the online Saccharomyces Genome Database (Cherry *et al*. 2012).

### Iron chelation assay

Iron chelation was quantified using a colorimetric assay as previously described (Santos *et al*. 2017). Three reagents were sequentially added to each well in a 96-well plate [20 μL of 0.3 mM FeCl_2_, 40 μL of 0.8 mM Ferrozine (Sigma), and 100 μL of compound]. Then, the mixture was incubated for 10 min at room temperature (RT), and the absorbance of each well was measured at 562 nm on an EnVision Plate Reader (PerkinElmer). Chelation activity was calculated using the following formula:


%chelationactivity=100*[(Ac−As/Ac)]

where *A*_c_ is the absorbance of control and *A*_s_ is the absorbance of treatment (EtOH-vescalagin).

### Atomic absorption spectroscopy

Intracellular levels of zinc and iron were quantified using atomic absorption spectroscopy (AAS) as previously described (Simm *et al*. 2007). In brief, cells were grown to mid-log in 1 mL SC, centrifuged at 1000×g for 5 min, washed twice in cold 0.5 mM EDTA, washed in cold deionized water, digested in nitric acid (40% *v*/*v*) overnight at 90°C on a heat block, and measured with standards (Sigma) on an AAS (iCE3500) instrument fitted with a GFS35 graphite furnace unit (Thermo Fisher Scientific) using argon as the purge gas.

### Protein expression

Abundance and localization of proteins were investigated in strains expressing a GFP-tagged protein of interest relative to internal RFP-tagged markers of the cytoplasm and nucleus (Bircham *et al*. 2011). Cells were grown to mid-log in SC in 96-well plates, transferred to a YPD agar plate in 384 format, and incubated at 30°C for 14 h. Cells from this plate were then transferred to SC media with and without EtOH-vescalagin in a black-walled, clear-bottom 384-well plate (PerkinElmer), incubated at 30°C for 4 h, and visualized using Evotec Opera high-throughput confocal microscope (PerkinElmer) with an exposure time of 400 ms and 3 stacks per field. GFP excitation was at 488 nM, while RFP (mCherry and RedStar) excitation was at 561 nm. GFP quantification was completed using ACAPELLA v2.0 (PerkinElmer) that recognized cells by the RFP markers (i.e. the cytosol with RedStar and the nucleus with mCherry). GFP expression was normalized against RFP expression for each well.

### Gene overexpression analysis

Plasmids expressing specific genes under the control of the GAL1 promoter (Cooper *et al*. 2006) were transformed in yeast strains using lithium acetate (Schiestl and Gietz 2007). Transformed strains were grown overnight in liquid SC–Ura + 2% raffinose, and then, growth was compared using the liquid growth assay in SC–Ura + 2% glucose relative to SC–Ura + 2% galactose + 1% raffinose.

### Zincosome analysis

Cytoplasmic zinc-containing vesicles (zincosomes) were visualized using the fluorescent stain zinquin [zinquin ethyl ester (2-methyl-8-[(4-methylphenyl)sulfonylamino]-6-(ethyloxycarbonylmethyloxy)quinolone); Sigma] as previously described (Devirgiliis *et al*. 2004). Cells were grown overnight in SC media and then inoculated at OD_600_ of 0.1 in SC media with and without EtOH-vescalagin. Cells at mid-log phase of growth were washed with PBS, fixed with 3.7% formaldehyde for 1 h at 30°C, washed with PBS, resuspended in 25 μM zinquin (in PBS), incubated for 30 min at RT, washed twice with PBS, imaged using fluorescent microscopy (Olympus BX63), and quantified using ImageJ.

## Results

### All feijoa polyphenols are bioactive in yeast

**Fig. 2.**
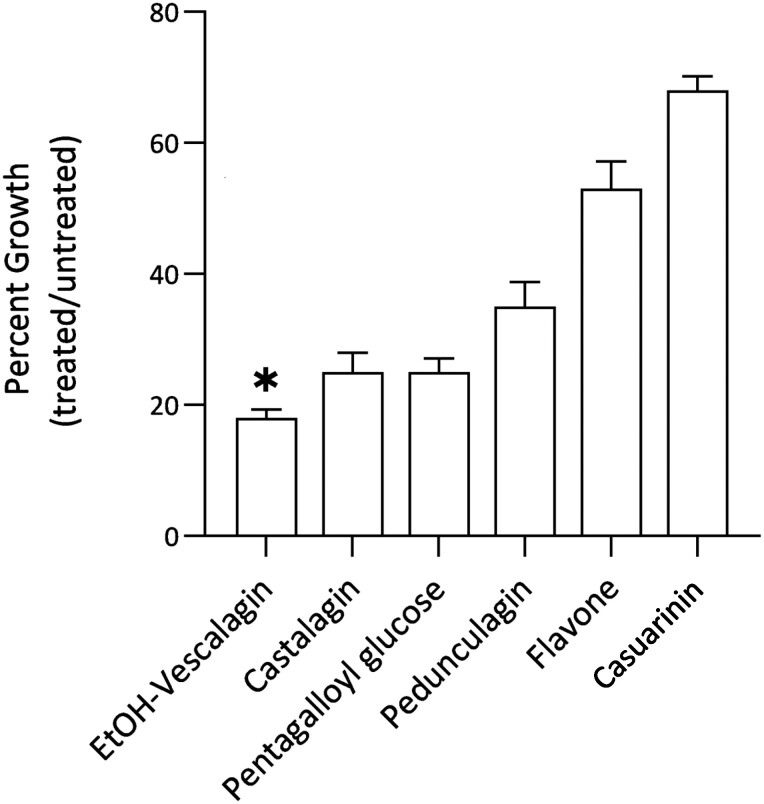
Feijoa-derived polyphenol compounds inhibit the growth of yeast. WT cells were cultured overnight, transferred to fresh media in the absence or presence of 1 µM of each compound, and incubated at 30°C until untreated cells reached mid-log. Data shown as mean ± SD of triplicate cultures; *, 2-tailed Student's *t* test comparing EtOH-vescalagin with each other compound.

**Fig. 3.**
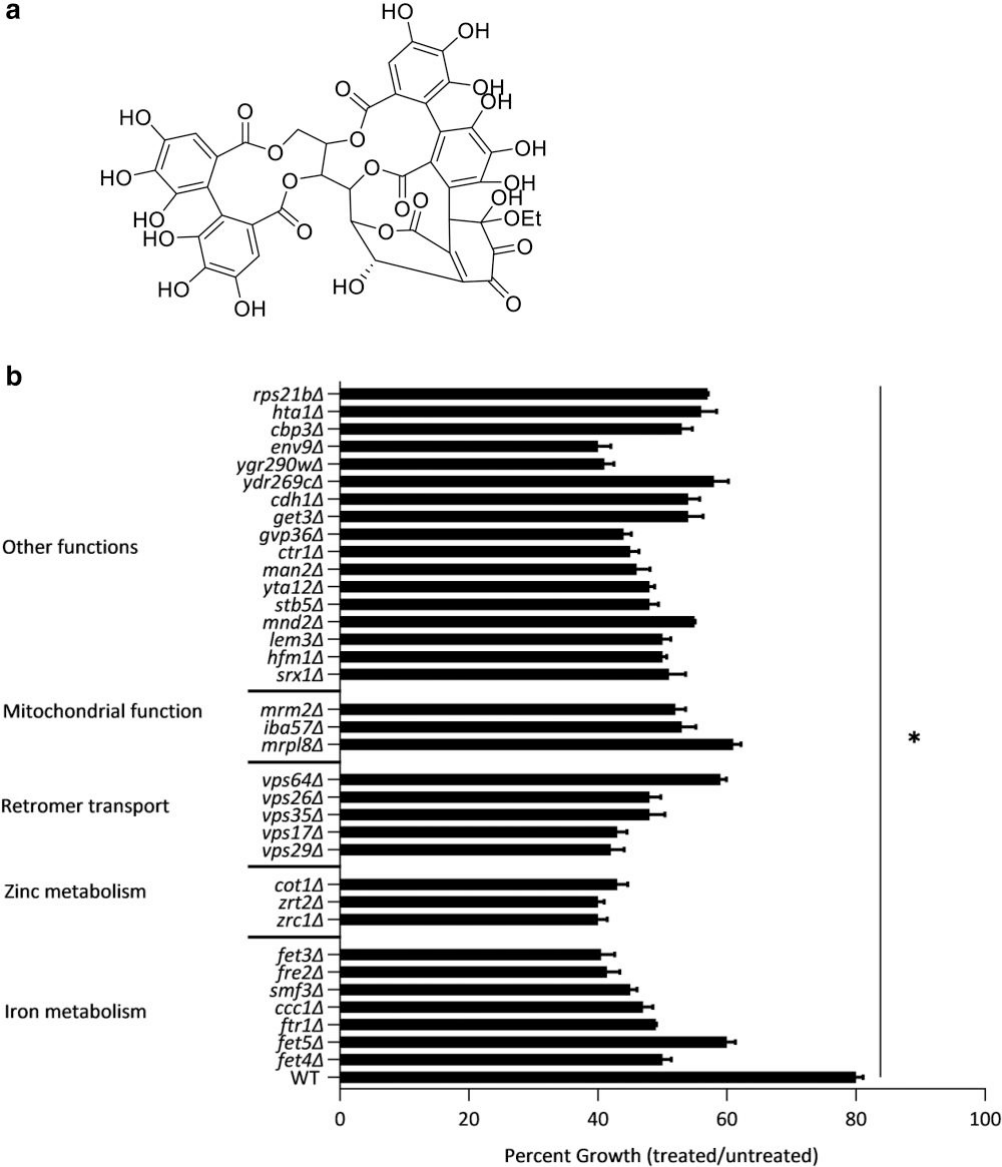
a) Structure of EtOH-vescalagin. b) Sensitivity of 36 gene deletion strains to EtOH-vescalagin. Each strain was cultured overnight, transferred to fresh media in the absence or presence of 0.3 µM EtOH-vescalagin, and incubated at 30°C until untreated cells reached mid-log. Data shown as mean ± SD of triplicate cultures; *, 2-tailed Student's *t* test for comparing each deletion strain relative to WT.

To initially evaluate bioactivity of the tannin compounds isolated from feijoa fruit, growth of wild-type (WT) yeast (BY4741) was quantified in the presence and absence of each compound, where reduced growth in treated cells indicates increased sensitivity (increased bioactivity). At 1 μM, all 6 polyphenol compounds inhibited growth and were bioactive (Fig. 2). EtOH-vescalagin showed highest activity with 82% inhibition, followed by castalagin and pentagalloyl glucose with 75% growth inhibition. Less potent were pedunculagin and casuarinin with 35 and 68% growth inhibition, respectively. For comparison, growth in the presence and absence of an equivalent concentration of flavone, an established bioactive polyphenol (Panche *et al*. 2016), was also assessed. Notably, flavone resulted in 53% growth inhibition relative to untreated control cells, further highlighting the bioactivity of EtOH-vescalagin. We thus chose to further investigate the mechanism of action of EtOH-vescalagin (Fig. 3a), the most potent compound isolated from feijoa fruit.

### Bar-seq analysis identifies key processes to EtOH-vescalagin bioactivity via sensitivity of gene deletion strains

As genome-wide Bar-seq analysis of gene deletion libraries serves as a well-established approach for elucidating the mechanisms driving the bioactivity of various compounds (Hillenmeyer *et al*. 2008; Lee *et al*. 2014), we next sought to conduct a Bar-seq analysis to elucidate the underlying mechanism for the bioactivity of EtOH-vescalagin. Pools of the homozygous diploid deletion mutant library were grown in the presence or absence of 0.3 µM EtOH-vescalagin, a concentration that inhibits the growth of WT by 20% (IC_20_) relative to untreated control, which provides a large 80% window to detect growth reduction due to gene deletions. Via the abundance of UPTAG and DNTAG barcodes flanking each gene deletion in a pooled condition (Supplementary Table 1) and replication in isolated deletion strains, 36 gene deletions associated with multiple biological processes resulted in increased sensitivity to EtOH-vescalagin (Fig. 3b). Deletion of iron transporter genes (*FTR1*, *FET3*, *FET4*, *FET5*, *SMF3*) as well as iron reductase genes (*FRE1*, *FRE2*) resulted in increased sensitivity to EtOH-vescalagin. Zinc metabolism was also implicated via hypersensitivity of zinc transporter deletions that transport zinc into the cell (ZRT2) and into the vacuole (ZRC1 and COT1). Third, the retromer complex was implicated via genes involved in the retromer recycling pathway (VPS5, VPS10, VPS17, VPS35, VPS26, VPS29, VPS64) that are responsible for retrograde endosome-to-Golgi transport. Lastly, mitochondrial function was implicated via sensitivity of genes integral to mitochondria function (IBA57, MRPL8, and MRM2). Overall, these results implicate iron and zinc homeostasis, retromer recycling, and mitochondrial function as possible mechanisms mediating the bioactivity of EtOH-vescalagin.

### Iron supplementation modulates EtOH-vescalagin bioactivity

**Fig. 4.**
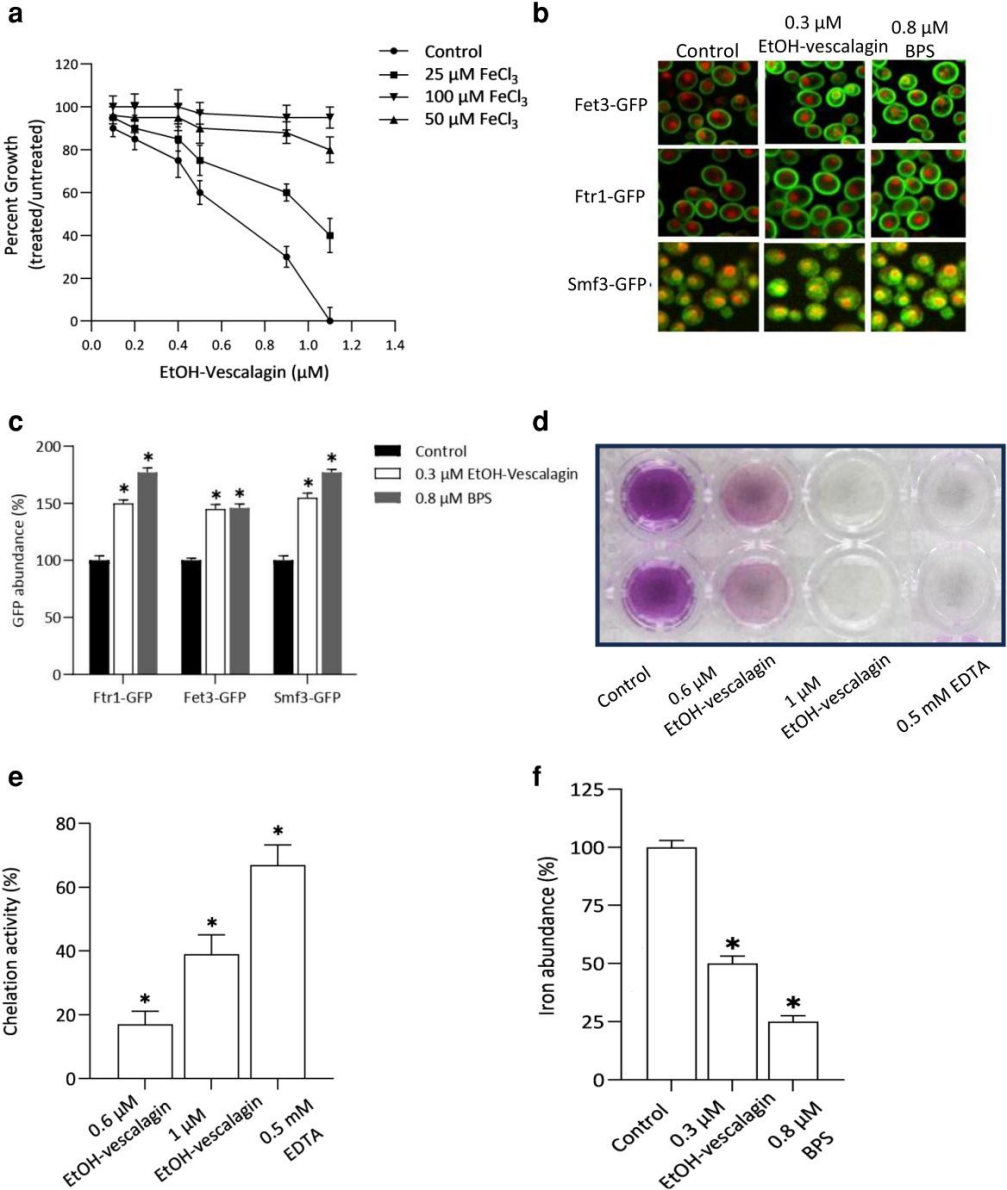
a) Iron supplementation reverses bioactivity of EtOH-vescalagin. WT cells were cultured overnight, transferred to fresh media in the absence or presence of increasing concentrations of EtOH-vescalagin with or without FeCl_3_, and incubated at 30°C until untreated cells reached mid-log. Data shown as mean ± SD of triplicate cultures. b) EtOH-vescalagin alters the abundance of GFP-tagged iron transporter proteins. Cells were cultured overnight, transferred to fresh media in the absence or presence of either 0.3 µM EtOH-vescalagin or 0.8 µM BPS, incubated at 30°C until untreated cells reached mid-log, and imaged at 60× using an Opera fluorescent microscope (PerkinElmer). c) Quantification of protein abundance for each condition in b). ACAPELLA was used to quantify GFP abundance relative to cytosolic/nuclear RFP; *n* ≥ 500 cells for each condition. *, 2-tailed Student's *t* test for comparing each treatment relative to control. d) EtOH-vescalagin chelates iron. Iron chelation activity of EtOH-vescalagin compared with EDTA was measured in a cell-free CAS assay. A color change from color to colorless is indicative of iron chelation. e) Quantification of iron chelation in d). Iron chelation was measured 10 min after treatment via absorbance. *, 2-tailed Student's *t* test for comparing each treatment relative to control. f) EtOH-vescalagin reduces intracellular levels of iron. Cells were cultured overnight, transferred to fresh media in the absence or presence of either 0.3 µM EtOH-vescalagin or 0.8 µM BPS, and incubated at 30°C until untreated cells reached mid-log, and furnace AAS was used to measure intracellular iron. Data shown as mean ± SD of triplicate cultures; *, 2-tailed Student's *t* test for comparing each treatment relative to control.

Given that the Bar-seq analysis identified iron transporter deletion strains sensitive to EtOH-vescalagin, we hypothesized EtOH-vescalagin treatment results in reduced intracellular iron levels, and if so, iron supplementation would rescue the inhibition. We thus quantified growth of WT cells in the presence of varying concentrations of FeCl_3_ and EtOH-vescalagin (Fig. 4a). As expected, increasing EtOH-vescalagin concentration resulted in increased amounts of growth inhibition (e.g. 72 and 100% growth inhibition with treatments of 0.9 and 1.1 µM EtOH-vescalagin, respectively). The supplementation of 25 μM FeCl_3_ partially rescued growth defects by 15–40%, and even more impressively, supplementation of either 50 or 100 μM FeCl_3_ completely rescued growth defects even in the case of the lethal concentration of 1.1 μM EtOH-vescalagin. Overall, restoration of growth by iron supplementation strengthens the notion that EtOH-vescalagin reduces intracellular iron content.

### EtOH-vescalagin increases expression of iron transporters

Given that iron supplementation rescued growth inhibition caused by EtOH-vescalagin (Fig. 3a), we predict a cell treated with EtOH-vescalagin will exhibit the molecular signatures of iron deficiency. Increased expression of iron transporters at the plasma membrane is an established compensatory response to iron deficiency (Philpott and Protchenko 2008). To assess expression of iron transporters, we exploited the yeast strains in the GFP library for which proteins are endogenously tagged with a GFP; thus, fluorescence reflects endogenous levels and localization. Relative to RFP fluorescence of nuclei marked with mCherry and the cytosol marked with RedStar2, we evaluated the abundance of high-affinity iron transporters at the plasma membrane (Fet3-GFP, Ftr1-GFP) and an iron transporter at the vacuolar membrane (Smf3-GFP) in response to IC_20_ treatments of either 0.3 µM EtOH-vescalagin or an iron chelator control (0.8 µM BPS). EtOH-vescalagin increased levels of Fet3-GFP, Ftr1-GFP, and Smf3-GFP by 38, 54, and 62%, respectively, compared with the untreated control (Fig. 4b and c). For comparison, the iron chelator BPS treatment increased levels of Fet3-GFP, Ftr1-GFP, and Smf3-GFP by 66, 41, and 31%, respectively, compared with the untreated control (Fig. 4b and c). With EtOH-vescalagin eliciting comparable cellular responses to that of the iron chelator BPS, these results provide additional evidence for EtOH-vescalagin reducing intracellular iron content of cells.

### EtOH-vescalagin chelates iron

The reversal of growth inhibition with iron supplementation and the similarity in altered expression of proteins with the iron chelator BPS suggest that EtOH-vescalagin chelates iron. To determine if EtOH-vescalagin chelates iron, we [the cell-free chrome azurol S (CAS) assay] tested the efficacy of EtOH-vescalagin to chelate iron from a complex of ferrozine bound to iron (Fig. 4d). The ferrozine–iron complex forms a purple color, which, upon treatment with the iron chelator control EDTA, turns into a clear solution in a dose-dependent manner. Likewise, EtOH-vescalagin chelates the iron from the ferrozine–iron complex in a dose-dependent manner. The chelation activity of EtOH-vescalagin was 16.5, 38.3, and 58.4% with treatments of 0.6 µM, 1 μM, and 1 mM, respectively, which was indistinguishable from the 19.8, 47.7, and 66.3% chelation activity with iron chelator treatments of 0.6 µM, 1 μM, and 1 mM EDTA, respectively (Fig. 4e). These results suggest that EtOH-vescalagin is as potent an iron chelator as EDTA and also indicate that the mechanism of action of EtOH-vescalagin begins with the chelation of iron in the media and proceeds with inducing a cellular response to low iron.

### EtOH-vescalagin decreases intracellular iron levels

Our results thus far suggest that EtOH-vescalagin reduces the availability of iron. To gain direct and quantitative insight into the effect of EtOH-vescalagin on cellular iron levels, we measured intracellular iron via AAS. Outstandingly, EtOH-vescalagin or BPS significantly reduced intracellular iron levels < 50% relative to the untreated control (Fig. 4f). These results confirm that EtOH-vescalagin reduces intracellular iron levels.

### Vacuolar storage of zinc buffers EtOH-vescalagin bioactivity

**Fig. 5.**
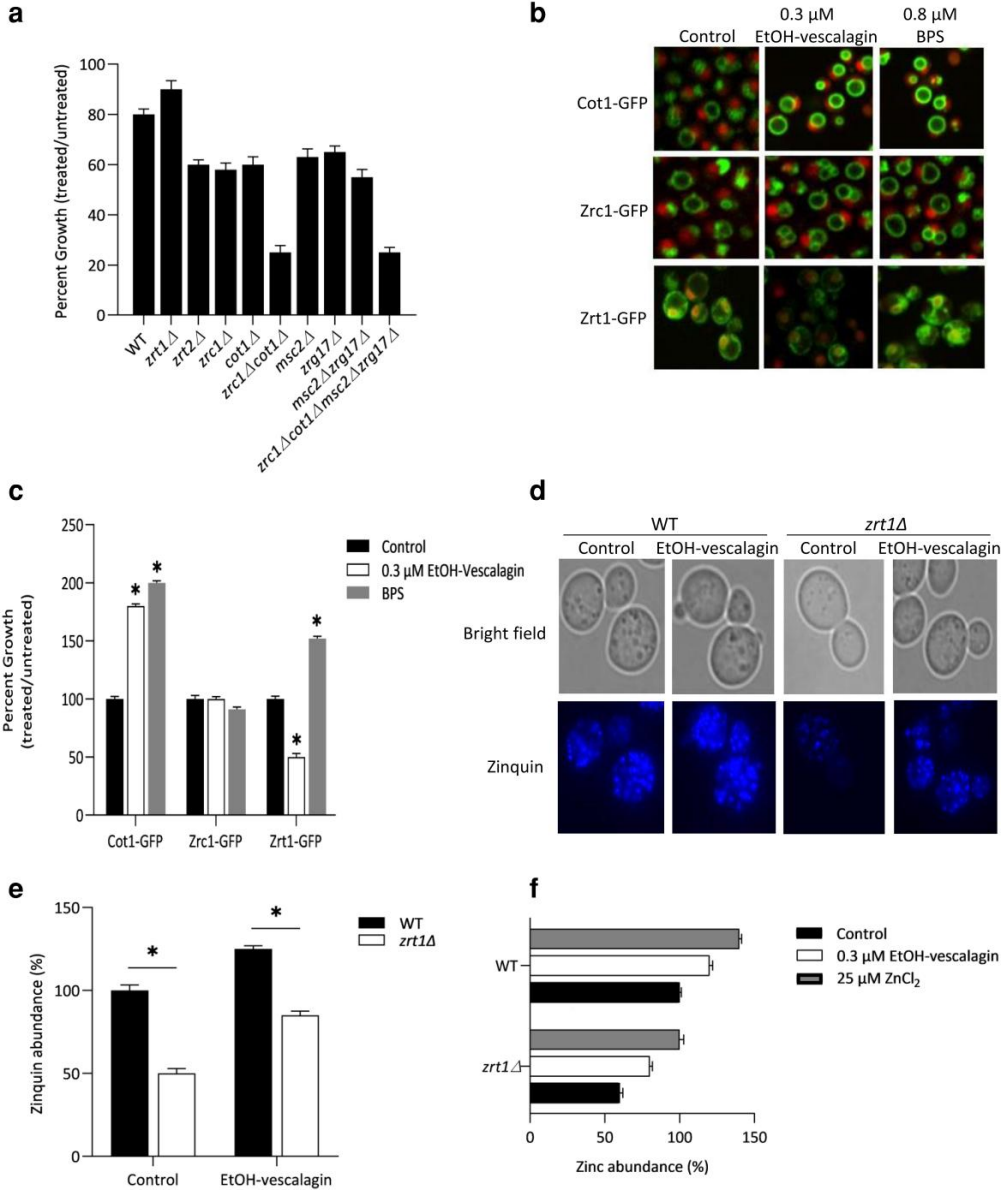
a) Sensitivity of zinc transporter deletion strains to EtOH-vescalagin. Each strain was cultured overnight, transferred to fresh media in the absence or presence of 0.3 µM EtOH-vescalagin, and incubated at 30°C until untreated cells reached mid-log. Data shown as mean ± SD of triplicate cultures; *, 2-tailed Student's *t* test for comparing each mutant relative to WT. 4Δ is quadruple deletion mutant (*zrc1Δcot1Δmsc2Δzrg17Δ*). b) EtOH-vescalagin alters the abundance of GFP-tagged zinc transporter proteins. Cells were cultured overnight, transferred to fresh media in the absence or presence of either 0.3 µM EtOH-vescalagin or 0.8 µM BPS, incubated at 30°C until untreated cells reached mid-log, and imaged at 60× using an Opera fluorescent microscope (PerkinElmer). c) Quantification of protein abundance for each condition in b). ACAPELLA was used to quantify GFP abundance relative to cytosolic/nuclear RFP; *n* ≥ 500 cells for each condition. *, 2-tailed Student's *t* test for comparing each treatment to control. d) EtOH-vescalagin increases abundance of zincosomes. Cells were cultured overnight, transferred to fresh media in the absence or presence of either 0.3 µM EtOH-vescalagin or 0.8 µM BPS, incubated at 30°C until untreated cells reached mid-log, stained with zinquin, and imaged at 100× using a fluorescent microscope. e) Quantification of zincosome abundance for each condition in d); *n* = 300 cells for each condition. f) EtOH-vescalagin increases intracellular levels of zinc. Cells were cultured overnight, transferred to fresh media in the absence or presence of either 0.3 µM EtOH-vescalagin or 25 µM ZnCl_2_, and incubated at 30°C until untreated cells reached mid-log, and furnace AAS was used to measure intracellular zinc. Data shown as mean ± SD of triplicate cultures; *, 2-tailed Student's *t* test for comparing each treatment relative to control.

Chemical genetic profiling thus far revealed that loss of either zinc transporters at the plasma membrane or vacuole resulted in greater sensitivity to EtOH-vescalagin (Fig. 3b). Zinc serves as a catalytic and structural cofactor for numerous proteins whereby critical to many biological processes. Zinc uptake occurs at the plasma membrane via the high-affinity transporter ZRT1 and the low-affinity transporter ZRT2, and then, intracellular zinc is transported into the vacuole by ZRC1 and COT1 or into the endoplasmic reticulum by MSC2 and ZRG17 (Eide 2006). We thus evaluated the contributions of these genes and associated compartments (Fig. 5a). Relative to WT, the loss of ZRT2 marginally increased sensitivity to EtOH-vescalagin, while the loss of ZRT1 exhibited marginally reduced sensitivity. The single deletions regulating vacuolar zinc transport (*zrc1Δ*, *cot1Δ*) were each sensitive to EtOH-vescalagin with ∼45% growth defect, while the *zrc1Δcot1Δ* double deletion exhibited greater sensitivity with a 76% growth defect. The single deletions regulating ER zinc transport (*msc2Δ*, *zrg17Δ*) were each sensitive to EtOH-vescalagin with ∼40% growth defect, while the *msc2Δzrg17Δ* double deletion did not exhibit any significant difference in EtOH-vescalagin sensitivity compared with the single deletions. Of note, the lack of all intracellular zinc transporters (*msc2Δzrg17Δzrc1Δcot1Δ*) did not further reduce the growth defect relative to *zrc1Δcot1Δ*, revealing that vacuolar storage of zinc is a critical mechanism to EtOH-vescalagin bioactivity.

### EtOH-vescalagin increases expression of zinc transporters at the vacuolar membrane

Increased expression of transporters importing zinc into the vacuole is an established means for a cell to store excess zinc in the vacuole (MacDiarmid *et al*. 2000). To assess expression of the proteins regulating import of zinc, we evaluated abundance of Zrc1-GFP and Cot1-GFP at the vacuolar membrane as well as the high-affinity zinc transporter Zrt1-GFP at the plasma membrane in response to EtOH-vescalagin (Fig. 5b and c). Relative to the untreated control, the Cot1-GFP was increased by 77% and the Zrt1-GFP was decreased by 48% upon treatment with EtOH-vescalagin. No change in expression was detected in the other vacuolar membrane transporter Zrc1-GFP, possibly since the increased expression of Cot1-GFP is the primary means of importing zinc into the vacuole. For comparison, the iron chelator BPS elicited a different effect as EtOH-vescalagin with increased expression of Cot1-GFP by 98% and Zrt1-GFP by 46% compared with untreated controls, results that are consistent with increased COT1 expression as a protective mechanism to sequester zinc in the vacuole in response to iron deficiency (Philpott and Protchenko 2008) or store zinc in response to zinc toxicity (Simm *et al*. 2007). These results indicate that increased expression of Cot1-GFP at the vacuolar membrane and decreased expression of Zrt1-GFP at the plasma membrane are mechanisms by which cells treated with EtOH-vescalagin respond to zinc toxicity.

### EtOH-vescalagin increases intracellular zincosomes

Besides the vacuole, zincosomes, which are zinc-enriched cytoplasmic vesicles, serve as storage sites for excess zinc (Devirgiliis *et al*. 2004; Eide 2006; Simm *et al*. 2007). Induction of the zinc vacuolar transporter, cot1, further argued in favor of an increase in intracellular zinc levels and prompted us to monitor the abundance of zincosomes. We used the fluorescent stain zinquin to visualize zincosomes (Fig. 5d). In WT cells, EtOH-vescalagin significantly increased zincosome abundance in WT as well as the *zrt1Δ* strain that has previously been recognized with reduced numbers of zincosomes (Yuan 2011). These results indicate that the increased intracellular zinc load caused by EtOH-vescalagin treatment is a consequence, at least partially, of an increase in zincosomes.

### EtOH-vescalagin increases intracellular zinc levels

To gain direct and quantitative insight into the effect of EtOH-vescalagin on cellular zinc levels, we measured intracellular zinc via AAS (Fig. 5f). The treatment of EtOH-vescalagin led to a comparable 18–20% increase in intracellular zinc levels in either WT or the zinc-deficient *zrt1Δ* strain, similar to cells supplemented with 25 μM ZnCl_2_. These results confirm that intracellular zinc accumulation contributes to the bioactivity of EtOH-vescalagin.

### EtOH-vescalagin bioactivity links the retromer complex with iron and zinc homeostasis

The retromer complex is a fundamental component of trafficking machinery that recycles proteins from endosomes to the *trans*-Golgi network or plasma membrane (Seaman 2021). The retromer complex is composed of 2 major subcomplexes that fulfill different roles: VPS26/VPS29/VPS35 mediates cargo recognition and VPS5/VPS10/VPS17 contributes to tubule and vesicle formation. Chemical genetic profiling has implicated the retrograde complex in mediating the bioactivity of EtOH-vescalagin, where mutations in either subcomplex (*vps17Δ*, *vps26Δ*, *vps29Δ*) exacerbate sensitivity to EtOH-vescalagin (Fig. 3b).

**Fig. 6.**
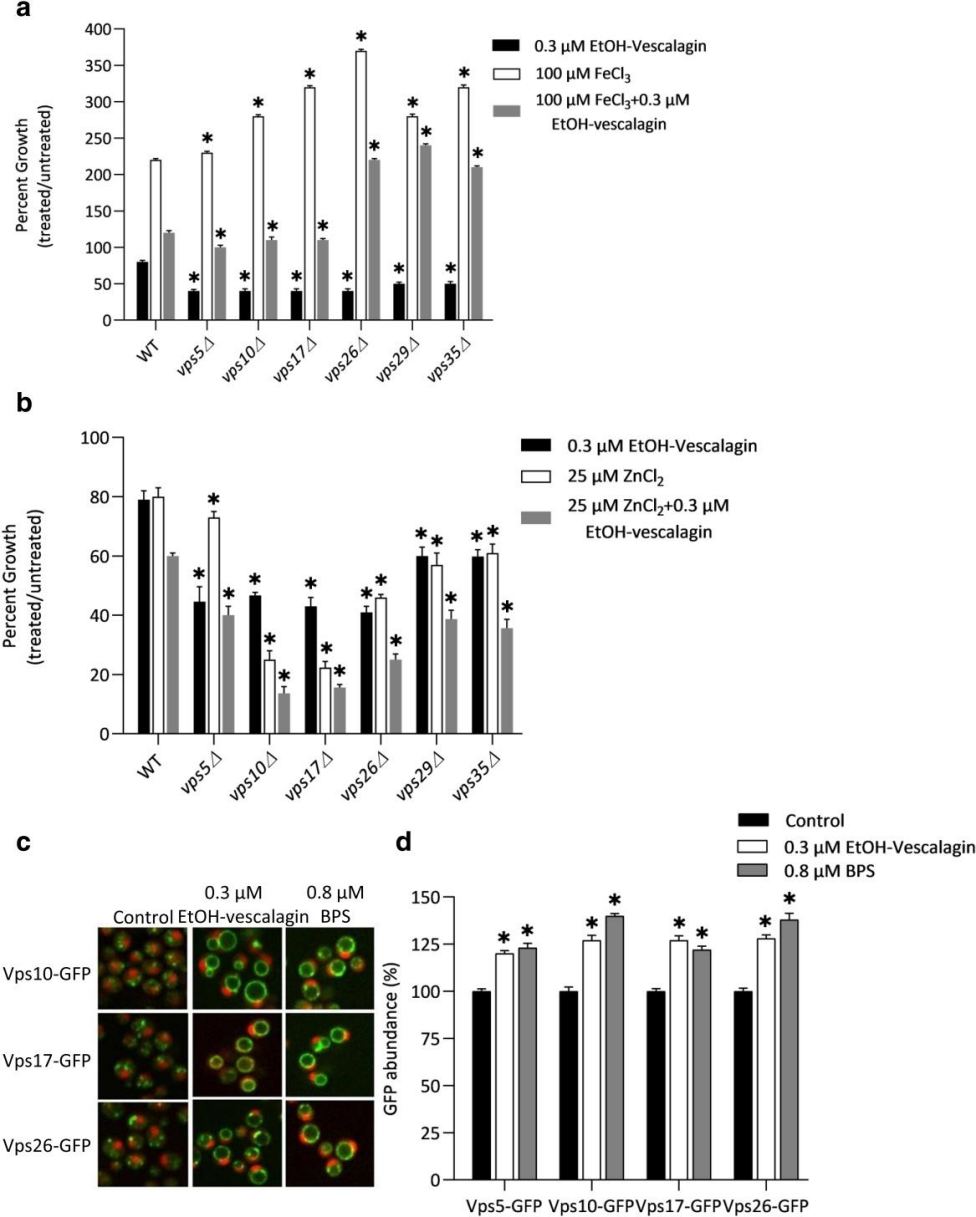
a) Sensitivity of retromer deletion strains to EtOH-vescalagin with and without iron. Each strain was cultured overnight, transferred to fresh media in the absence or presence of various combinations of 0.3 µM EtOH-vescalagin and 100 µM FeCl_3_, and incubated at 30°C until untreated cells reached mid-log. Data shown as mean ± SD of triplicate cultures; *, 2-tailed Student's *t* test for comparing each mutant relative to WT. b) Sensitivity of retromer deletion strains to EtOH-vescalagin with and without iron. Each strain was cultured overnight, transferred to fresh media in the absence or presence of various combinations of 0.3 µM EtOH-vescalagin and 25 µM ZnCl_2_, and incubated at 30°C until untreated cells reached mid-log. Data shown as mean ± SD of triplicate cultures; *, 2-tailed Student's *t* test for comparing each mutant relative to WT for each condition. c) EtOH-vescalagin alters the abundance of GFP-tagged retromer proteins. Cells were cultured overnight, transferred to fresh media in the absence or presence of either 0.3 µM EtOH-vescalagin or 0.8 µM BPS, incubated at 30°C until untreated cells reached mid-log, and imaged at 60× using an Opera fluorescent microscope (PerkinElmer). d) Quantification of protein abundance for each condition in b). ACAPELLA was used to quantify GFP abundance relative to cytosolic/nuclear RFP; *n* > 500 cells for each condition.

To test the hypothesis that the biological significance of the retromer complex in EtOH-vescalagin bioactivity might be mediated via iron and/or zinc metabolism, growth of all gene deletions representing each subcomplex was measured in various combinations of EtOH-vescalagin and each metal (Fig. 6a and b). Notably, all single retromer gene deletions were more sensitive to EtOH-vescalagin than WT, which further strengthened our finding that retromer transport is a mechanism in EtOH-vescalagin bioactivity. The impacts of iron and zinc were not the same. While iron supplementation alone improved growth of all strains, EtOH-vescalagin treatment suppressed this bioactivity, and this suppression was least apparent in *vps29Δ* and *vps35Δ*. In contrast, only deletion strains in the VPS5/VPS10/VPS17 complex were sensitive to zinc supplementation, and zinc supplementation either phenocopied EtOH-vescalagin treatment (WT, *vps26Δ*, *vps29Δ*, *vps35Δ*), suppressed EtOH-vescalagin treatment (*vps5Δ*), or exacerbated EtOH-vescalagin treatment (WT, *vps10Δ*, *vps17Δ*). Owing to the increased sensitivity of *vps10Δ* and *vps17Δ* to zinc supplementation independent of EtOH-vescalagin, it is plausible that disruption of the retromer complex leads to an increase in intracellular zinc level thereby causing hypersensitivity to EtOH-vescalagin that also increases intracellular zinc levels. Overall, the retromer mutants were variably sensitive to iron and zinc, which EtOH-vescalagin further modified this sensitivity.

### EtOH-vescalagin disrupts protein abundance in the retromer complex

We further investigated the contribution of the retromer complex to EtOH-vescalagin bioactivity via monitoring the abundance and localization of GFP-tagged retromer proteins in the presence of EtOH-vescalagin (Fig. 6c and d). Relative to the untreated control, 0.3 μM EtOH-vescalagin increased the abundance of Vps10-GFP, Vps17-GFP, and Vps26-GFP by 17, 27, 24, and 28%, respectively, compared with untreated cells. In addition to increased cellular levels, the EtOH-vescalagin treatment altered the localization of these Vps10-GFP, Vps17-GFP, and Vps26-GFP from endosomes to the vacuolar membrane. We also evaluated the levels of these proteins in response to the iron chelator BPS to further discern the effects of iron chelation on the retromer complex. The 0.8 μM BPS treatment phenocopied the EtOH-vescalagin treatment with increased expression and relocalization from endosomes to vacuolar membrane. These results are consistent with the relocalization of Vps10-GFP from endosomes to the vacuolar membrane upon disruption of retromer function (Burda *et al*. 2002), suggesting that this relocalization is an adaptive physiological response to iron-chelating bioactivity of EtOH-vescalagin.

### Overexpression of iron/zinc transporters and retromer subunits modulates sensitivity to EtOH-vescalagin

**Fig. 7.**
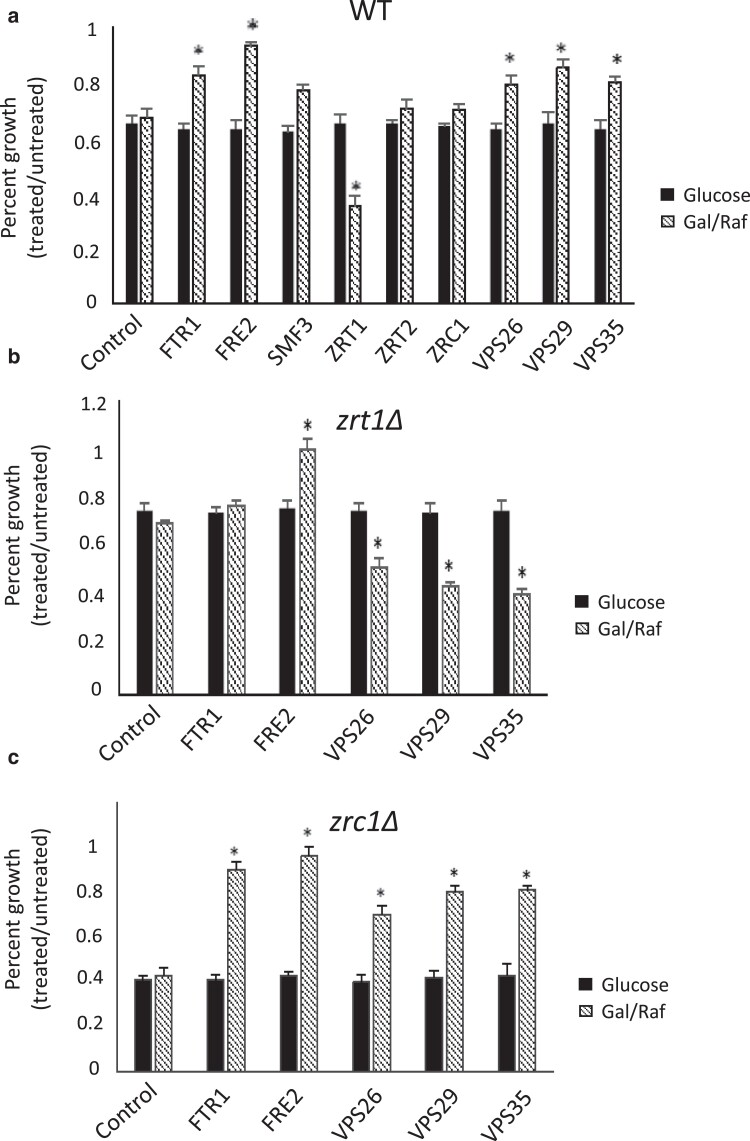
Overexpression of iron transporters, zinc transporters, and retromer subunits alters the sensitivity of a) WT, b) zinc-deficient *zrt1Δ*, and c) zinc-replete *zrc1Δ*. Each strain expressing an empty vector or gene was cultured overnight in SC–Ura + raffinose, transferred to fresh media to induce overexpression (SC–Ura + galactose/raffinose) or repress overexpression (SC–Ura + glucose) in the absence or presence of 0.3 µM EtOH-vescalagin, and incubated at 30°C until untreated cells reached mid-log. Data shown as mean ± SD of triplicate cultures; *, 2-tailed Student's *t* test for comparing each gene overexpression relative to empty vector.

Our results thus far reveal that iron chelation and zinc toxicity and retromer complex disruption are underlying mechanisms for the bioactivity of EtOH-vescalagin. To further investigate this tripartite mechanism, we overexpressed iron transport (FTR1, FRE2, SMF3), zinc transport (ZRT1, ZRT2, ZRC1), and retromer (VPS26, VPS29, VPS35) genes in WT and assessed the growth in the presence and absence of EtOH-vescalagin on media either inducing overexpression (galactose) or repressing overexpression (glucose) (Fig. 7a). The overexpression of iron transporters (FTR1, FRE2) or retromer subunits (VPS26, VPS29, VPS35) suppressed growth inhibition by EtOH-vescalagin. In contrast, the overexpression of ZRT1 at the plasma membrane exacerbated growth inhibition by EtOH-vescalagin. These results largely complement growth inhibition previously observed with iron transport and retromer gene deletions (Fig. 3b), while overexpression of the high-affinity zinc transporter ZRT1 supports our model that EtOH-vescalagin induces zinc toxicity that is exacerbated with increased uptake of zinc from the media.

To further investigate the iron–zinc–retromer mechanism, we evaluated overexpression of iron transporters and retromer subunits in the zinc-deplete *zrt1Δ* strain (Fig. 7b) and the zinc-replete *zrc1Δ* strain (Fig. 7c). Overexpression of retromer subunits exacerbated growth inhibition by EtOH-vescalagin in *zrt1Δ*, while overexpression of the same retromer genes suppressed growth inhibition by EtOH-vescalagin in *zrc1Δ*. This result suggests that the retromer aspect of the mechanism is dependent on zinc homeostasis. In cases of both the zinc-deficient and zinc-replete conditions, overexpression of FRE2 suppressed growth inhibition by EtOH-vescalagin, which would be consistent with iron supplementation rescuing growth inhibition (Fig. 4a) and the iron aspect of the mechanism being upstream of zinc and retromer aspects.

## Discussion

This study aimed to determine the mechanism of action of EtOH-vescalagin. Using unbiased genome-wide Bar-seq analysis, we identified yeast strains lacking genes in iron metabolism, zinc metabolism, retromer function, or mitochondrial function were hypersensitive to EtOH-vescalagin. The EtOH-vescalagin treatment resulted in iron deficiency in the cell based on sensitivity of iron transporter deletion strains, upregulation of iron transporters, rescued growth reduction with iron supplementation, and measurements of intracellular iron. Subsequent to iron chelation, there was an increase in intracellular zinc, and each individual subunit in the Vps26/Vps29/Vps35 retromer complex was required for the iron and zinc homeostatic mechanisms of EtOH-vescalagin.

Our study is the first report for iron chelation as a mechanism for vescalagin or EtOH-vescalagin. Since we showed that EtOH-vescalagin chelated iron in a cell-free assay and that the iron chelation activity of EtOH-vescalagin was indistinguishable from the known extracellular iron chelator BPS, we propose that EtOH-vescalagin chelates iron in the media, thus inducing the cellular response to iron deficiency. Iron chelation by plant polyphenols is an established mechanism underlying a range of bioactivities (Phiwchai *et al*. 2018). Relative to our study where we demonstrated antifungal activity of EtOH-vescalagin via growth inhibition of yeast, iron chelation has been proposed as a mechanism for antifungal treatment where defects in the high-affinity iron uptake system significantly enhanced antifungal activity of clinically approved antifungal drugs against pathogenic fungi including *C. albicans*, *Cryptococcus neoformans*, and *Aspergillus fumigatus* (Kuipers *et al*. 1999; Zarember *et al*. 2009; Lai *et al*. 2016). This was consistently demonstrated with various iron-chelating agents such as EDTA, deferoxamine, deferiprone, deferasirox, ciclopirox olamine, and lactoferrin. Our results may provide mechanistic insight into the hypothesis that vescalagin in oak heartwood is one of the compounds combating the growth of pathogenic fungi in casks (Wakamatsu *et al*. 2020).

Since several zinc-regulated genes are also regulated by iron deprivation (Waters and Eide 2002; Santos *et al*. 2003), it is likely that EtOH-vescalagin increases intracellular levels of zinc via iron chelation. The Cot1-GFP transporter of zinc at the vacuolar membrane is increased in abundance with EtOH-vescalagin or iron chelation with BPS. FET4 is capable of transporting both iron and zinc and regulated by an iron transcription factor (AFT1) as well as a zinc transcription factor (ZAP1) (Eide 1998; Rutherford and Bird 2004). It has been demonstrated that iron deprivation, resulting from a defect in the FET3  ferroxidase required for high-affinity iron uptake, led to the upregulation of FET4 and increased uptake of zinc (Li and Kaplan 1998). Since we show FET4 is required to buffer EtOH-vescalagin bioactivity, it is plausible that EtOH-vescalagin upregulates FET4 and allows for excessive uptake of zinc.

Alternatively, since the *zrc1Δcot1Δ* strain lacking zinc transporters at the vacuolar membrane was the most sensitive strain to EtOH-vescalagin and overaccumulation of zinc triggers increased expression of the vacuolar zinc transporter COT1 that conversely downregulates the high-affinity plasma membrane zinc transporter ZRT1 (Eide 2006), it is plausible that the primary mode of action of EtOH-vescalagin is increasing intracellular zinc levels, which may secondarily reduce iron levels. Consistently, ZRT1 deficiency suppressed inhibitory effects of EtOH-vescalagin, presumably due to reduced uptake of zinc.

Overall, our results suggest an interplay between iron, zinc, and retromer complex in mediating the antifungal activity of EtOH-vescalagin. While EtOH-vescalagin sensitivity was reduced in retromer deletion strains with iron supplementation or overexpression of iron transporters at the plasma membrane, EtOH-vescalagin sensitivity was exacerbated in retromer deletion strains with zinc supplementation. EtOH-vescalagin sensitivity was also exacerbated with overexpression of retromer subunits in *zrt1Δ* (low intracellular zinc), while sensitivity was reduced with overexpression of retromer subunits in *zrc1Δ* (high intracellular zinc). As we show *vps10Δ* and *vps17Δ* are each sensitive to zinc supplementation, it is plausible that these components of the retromer complex are crucial for maintaining zinc homeostasis, which would be consistent with the report of an interaction between the sorting nexin SNX27 and the retromer complex that is integral to localization of the ZnT1 protein (the Zrc1/Cot1 ortholog) in human cells (Steinberg *et al*. 2014). Further investigation will add to our immature understanding of the zinc–retromer interaction (e.g. determining the molecular mechanisms behind the change in localization of Vps10 and Vps17 in response to EtOH-vescalagin treatment or zinc supplementation. Since there is a zinc requirement for mammalian VPS10-dependent phosphatase activity (Damen *et al*. 2006) and EtOH-vescalagin mislocalized yeast Vps10-GFP from the endosome to the vacuolar membrane in WT but not the zinc-deficient *zrt1Δ* strain, and this was phenocopied with the BPS iron chelator, it is possible that both iron and zinc homeostases are integral to proper function of the retromer complex.

In conclusion, the Bar-seq analysis and the yeast model overall were fundamental to developing our model for the mechanism of action of EtOH-vescalagin that begins with extracellular iron chelation and proceeds with zinc toxicity and defective transport of proteins due to disruptions in retromer and mitochondrial transport. As mitochondria require iron for several enzymes (Fe–S cluster, heme, electron transport chain) (Ward and Cloonan 2019), the iron chelation mechanism of EtOH-vescalagin may help explain our observation that the loss of mitochondrial genes exacerbated the growth-inhibiting bioactivity of EtOH-vescalagin, particularly since iron deficiency–mediated oxidative stress can induce zinc uptake (Santos *et al*. 2003). Our results showed EtOH-vescalagin is chelating iron to the same extent as BPS and impacting mostly the same pathways; however, there were some distinctions (e.g. zinc accumulation was unique to EtOH-vescalagin), which imply that antifungal activity of EtOH-vescalagin is not a consequence of iron chelation alone. It is plausible that other ellagitannins (e.g. candelitannin and tellimagrandin II that have also exhibited antifungal activity (Yamaguchi *et al*. 2011; Lipińska *et al*. 2014) also increase intracellular zinc levels and disrupt retromer recycling; however, no studies other than our study herein have simultaneously examined iron chelation, zinc homeostasis and retromer recycling.

## Supplementary Material

jkae098_Supplementary_Data

## Data Availability

All data reported herein are available in this manuscript. Supplemental material available at G3 online.
