# Supplementary material for: Chemical genomic analysis reveals the interplay between iron chelation, zinc homeostasis, and retromer function in the bioactivity of an ethanol adduct of the feijoa fruit–derived ellagitannin vescalagin
Source: G3 (Bethesda). 2024 May 28;14(7):jkae098. doi: 10.1093/g3journal/jkae098 (PMC11228861; doi:10.1093/g3journal/jkae098)
Supplement: jkae098_Supplementary_Data [file jkae098_supplementary_data.zip › Supplemental_Material_Legends_G3-2024-405053.docx]

**Supplementary files**

**Supplementary Table 1. The complete dataset of the Bar-seq analysis of EtOH-vescalagin.** Annotation for each ORF (open reading frame) and gene included logFC (log fold-change in barcode abundance in treated relative to untreated condition), logCPM (log of counts per million reflecting sequencing quality), LR (likelihood ratio reflecting difference in growth in treated relative to untreated condition), and FDR (False Discovery Rate).
